# Supplementary material for: A taxonomically and geographically constrained information base limits non-native reptile and amphibian risk assessment: a systematic review
Source: PeerJ. 2018 Nov 8;6:e5850. doi: 10.7717/peerj.5850 (PMC6230440; doi:10.7717/peerj.5850)
Supplement: Supplemental Information 2 [file peerj-06-5850-s002.docx]

Supplementary material S1 (expanded methods and additional figures)

Searches were conducted on the ISI Web of Science (3rd March 2016) using the following criteria: Topic = alien OR invasive OR non-native OR exotic OR non-indigenous OR feral AND Topic= reptil* OR amphibia* OR turtle* OR tortoise* OR lizard* OR herpetofauna OR crocod* OR anura OR caudata OR testudin* OR ophidia OR sauria OR squamata OR snake* OR frog* OR toad* OR salamand* OR newt*. This search resulted in 3135 papers. Many of these papers were not relevant to the current study and the results were further refined by excluding papers from the following disciplines (research areas): Anesthesiology ; Archaeology ; Arts Humanities Other Topics ; Audiology Speech Language Pathology; Automation Control Systems ; Biophysics ; Biotechnology Applied Microbiology ; Business Economics ; Cardiovascular System Cardiology ; Cell Biology ; Chemistry ; Computer Science ; Dentistry Oral Surgery Medicine ; Dermatology ; Emergency Medicine ; Engineering ; Entomology ; Fisheries ; Food Science Technology ; Gastroenterology Hepatology ; General Internal Medicine ; Geology ; Geriatrics Gerontology ; Health Care Sciences Services ; Immunology ; Information Science Library Science ; Medical Informatics ; Medical Laboratory Technology ; Meteorology Atmospheric Sciences ; Neurosciences Neurology ; Nutrition Dietetics ; Obstetrics Gynecology ; Oncology ; Ophthalmology ; Optics ; Orthopedics ;Pediatrics ; Pharmacology Pharmacy ; Physics ; Plant Sciences ; Psychology ; Public Environmental Occupational Health ; Radiology Nuclear Medicine Medical Imaging ; Research Experimental Medicine ; Robotics ; Sport Sciences ; Surgery ; Telecommunications ; Transplantation ; Urology Nephrology


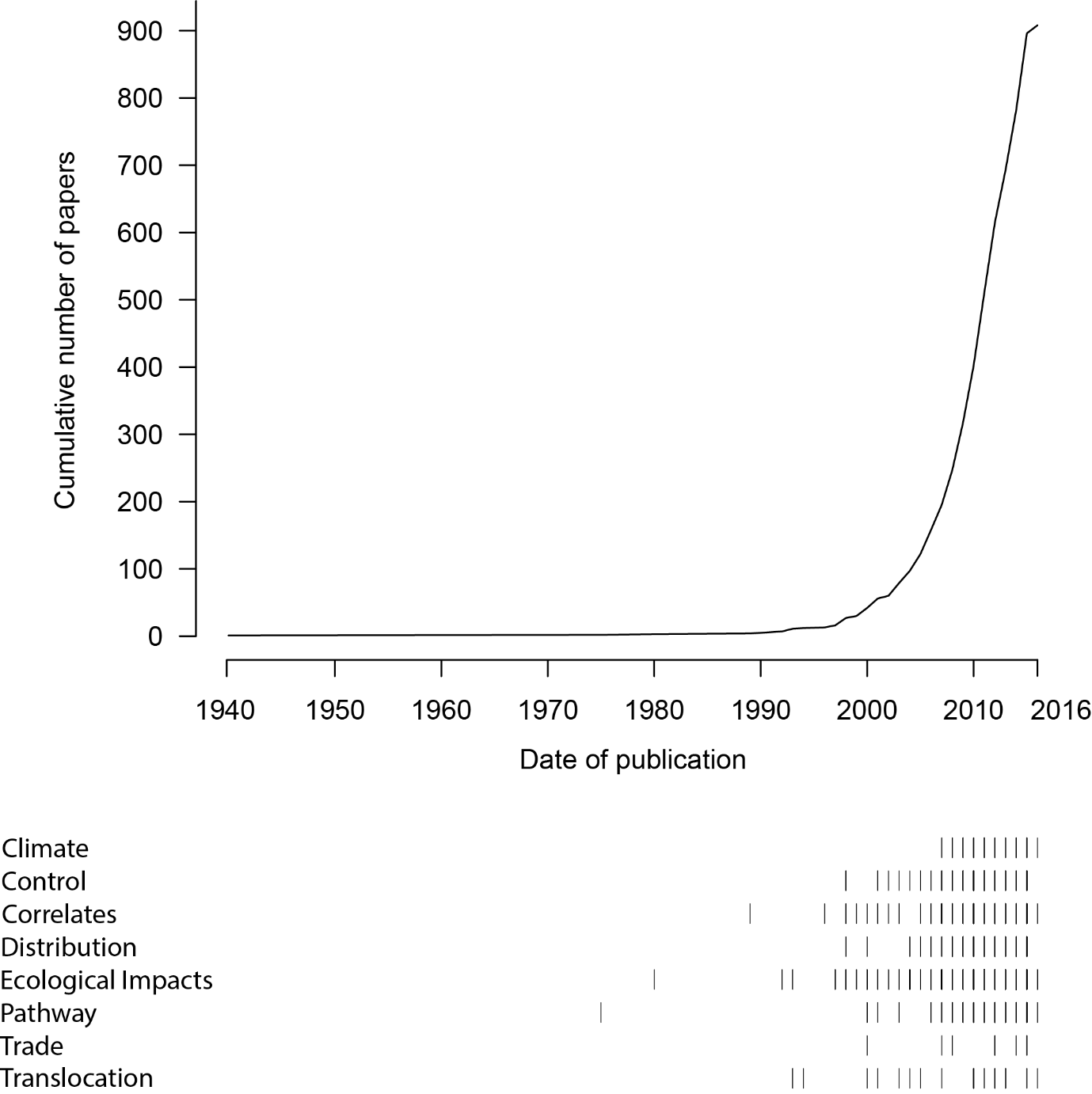


**Supplementary Fig. S1.** Cumulative trend in studies of non-native amphibians and reptiles for all taxa indicates the initial lag in studies up to the start of the 21th century. Data for each of the component groups demonstrates the different trends in invasive reptile and amphibian studies.

Supplementary Material S2

Details of the 836 papers included in the systematic review and the species recorded within each of these papers, as well as the geographic scope (at country level) of the papers are available as a supplementary Excel spreadsheet.
